# Wisconsin Dental Law

**Published:** 1885-08

**Authors:** 


### Wisconsin Dental Law.

Below we give the law regulating the practice of dentistry, recently enacted in the State of Wisconsin. This in its main features is similar to such laws in other States. It will be noticed in this, however, that all persons in it who seek to begin the practice of dentistry in the State, shall have the license of the examining board, this is required even of those holding diplomas from dental colleges.

The board is granted by law the power to judge of the standing of the colleges whose diplomas it will accept. This feature should be embraced in every such law; several laws however have it in an implied form, but it should be clearly and distinctly upon the face of every law.

The board of examiners appointed under this law consists of the following persons:

Dr. C. C. Chittenden, of Madison, five years; Dr. B. G. Marklin, Milwaukee, four years; Edgar Palmer, LaCross, three years; T. S. Reynolds, Monroe, two years; E. C. French, Eau Claire, one year. These men are well and favorably known to the profession and will undoubtedly carry out the provisions of the law most faithfully and secure by that means great benefit to the public and somewhat of advantage to the profession. We shall look for quite a revolution in the dental profession in the State of Wisconsin within the next five years.—[*Editor Register.*]

An Act to regulate the practice of dentistry, and to establish a State Board of Dental Examiners.

The people of the State of Wisconsin, represented in Senate and Assembly do enact as follows:

Section 1. It shall be unlawful for any person who is not, at the time of the passage of this act, engaged in the practice of dentistry in this State, to commence such practice, unless he shall have obtained a license as hereinafter provided.

Sec. 2. A board of examiners, to consist of five practicing dentists, is hereby created whose duty it shall be to carry out the purposes and enforce the provisions of this act. The members of said board shall be appointed by the Governor. Three members of this board, at least, shall be members of the Wisconsin State Dental Society. The terms for which the members of said board shall hold their offices shall be five years, except that the members of the board first to be appointed under this act shall hold their offices for the terms of one, two, three, four, and five years rspectively, and until their successors are appointed and qualified. In case of vacancy occurring in said board such vacancy shall be filled by the Governor.

SEC. 3. Said board shall choose one of its members President, and one Secretary thereof, and it shall meet at least once in each year and as much oftener and at such times and places as it may deem necessary. A majority of said board shall at all meetings constitute a quorum, and the proceedings thereof shall, at all reasonable times, be open to public inspection.

SEC. 4. It shall be the duty of every person who is engaged in the practice of dentistry in this State, within six months from the date of the passage of this act, and annually thereafter, to cause his or her name and residence or place of business to be registered with said board of examiners, who shall keep a book for that purpose; and every person who shall register with said board as a practitioner of dentistry may continue to practice the same as such without incurring any of the liabilities or penalities provided in this act. The board of examiners shall furnish to the county clerks a certified list of those registered and it shall be the duty of the county clerks to register such names in a book kept for such purpose. Every person registering with the board of examiners shall pay as a fee therefor the sum of one dollar.

SEC. 5. Any and all persons, who shall so desire, may appear before said board at any of its regular meetings and be examined with reference to their knowledge and skill in dental surgery, and if the examination of any such person or persons shall prove satisfactory to said board, the board of examiners shall issue to such persons, as they shall find from such examination to possess the requisite qualifications, a license to practice dentistry, in accordance with the provisions of this act. But said board shall at all times issue a license to any regular graduate of any reputable, legally incorporated dental college, which requires that the candidate for graduation shall attend two full courses of lectures of five months each, the last of which shall be attended in the institution granting the diploma, without examination, upon the payment by such graduate to the said board of a fee of one dollar. All licenses issued by said board shall be signed by the members thereof, and be attested by its President and Secretary; and such license shall be prima facie evidence of the rights of the holder to practice dentistry in the State of Wisconsin.

Sec. 6. Any person who shall violate any of the provisions of this act shall be liable to prosecution before any court of competent jurisdiction, upon information or by indictment, and upon conviction may be fined not less than fifty dollars nor more than two hundred dollars for each and every offense.

Sec. 7. In order to provide the means for carrying out and maintaining the provisions of this act, the said board of examiners may charge each person applying to or appearing before them for examination for license to practice dentistry, a fee of ten dollars. And out of the funds coming into their possession, from the fees mentioned in this act, the members of said board may receive all legitimate and necessary expenses incurred in attending the meetings of said board and in conducting the business thereof. Said expenses shall be paid from the fees received by the board, under the provisions of this act, and no part of the expenses of said board shall be paid out of the State treasury. All moneys received in excess of said expenses, above provided for, shall be held by the Secretary of said board as a special fund for meeting the expenses of said board, he giving such bond as the board shall from time to time direct. And said board shall make an annual report of its proceedings to the Governor, on the thirtieth day of September in each year, together with an account of all moneys received and disbursed by them pursuant to this act.

Sec. 8. This act shall take effect and be in force from and after its passage and publication.

Approved March 23, 1885.

